# Anomalous Small Angle X-Ray Scattering Simulations: Proof of Concept for Distance Measurements for Nanoparticle-Labelled Biomacromolecules in Solution

**DOI:** 10.1371/journal.pone.0095664

**Published:** 2014-04-23

**Authors:** Valerie J. Pinfield, David J. Scott

**Affiliations:** 1 Chemical Engineering Department, Loughborough University, Loughborough, Leicestershire, United Kingdom; 2 National Centre for Macromolecular Hydrodynamics, School of Biosciences, University of Nottingham, Sutton Bonington, Leicestershire, United Kingdom; 3 ISIS Neutron and Muon Spallation Source and Research Complex, Rutherford Appleton Laboratory, Harwell, Oxfordshire, United Kingdom; University of Leeds, United Kingdom

## Abstract

Anomalous small angle X-ray scattering can in principle be used to determine distances between metal label species on biological molecules. Previous experimental studies in the past were unable to distinguish the label-label scattering contribution from that of the molecule, because of the use of atomic labels; these labels contribute only a small proportion of the total scattering signal. However, with the development of nanocrystal labels (of 50–100 atoms) there is the possibility for a renewed attempt at applying anomalous small angle X-ray scattering for distance measurement. This is because the contribution to the scattered signal is necessarily considerably stronger than for atomic labels. Here we demonstrate through simulations, the feasibility of the technique to determine the end-to-end distances of labelled nucleic acid molecules as well as other internal distances mimicking a labelled DNA binding protein if the labels are dissimilar metal nanocrystals. Of crucial importance is the ratio of mass of the nanocrystals to that of the labelled macromolecule, as well as the level of statistical errors in the scattering intensity measurements. The mathematics behind the distance determination process is presented, along with a fitting routine than incorporates maximum entropy regularisation.

## Introduction

Small angle X-ray scattering of proteins and nucleic acids has enjoyed a recent renaissance due to improvements in instrumentation, analysis methods and computational processing speed [1]–[2]. The result is that SAXS, as a method of analysing macromolecular solution conformation and assemblies, has broadened from a few specialist laboratories and into the hands of a widening circle of users. With this resurgence has been a renewed interest in the technique of anomalous small angle X-ray scattering (ASAXS), where metal ions in a protein or nucleic acid complex alter the scattering pattern at wavelengths close to the absorbance edge of the ion [3]. Previously, such information has been used, in principle, to estimate distances between metal ions, such as the four iron atoms at the binding sites of haemoglobin [4]. The use of intrinsic metal binding sites of molecules has generally confined the biological applications of ASAXS to a single type of metal ion (in atomic form) attached to each binding site [5]. In addition, the weakness of the scattering signature from the ions relative to the whole molecule meant that only extremely limited information could be extracted about their location [4], [6]. In order to distinguish more information on the distance between specific sites it is desirable to use stronger scatterers and more than one ion type [7]. Metal labelling of biological material, especially using nanoclusters, can now be attained through standard chemistries, and therefore there is now the possibility of attaching multiple labels to a protein or nucleic acid. ASAXS could then potentially be used to determine the distances between similar and dissimilar metal types. Theoretically these distances could be several hundred nanometers, which is an order of magnitude better than the alternative distance-measurement technique, Fluorescence Resonance Energy Transfer (FRET), where the maximum distances are around 10 nm. However, the theory of ASAXS in such situations is currently underdeveloped and the feasibility of the technique as a ‘ruler’ has not yet been established. This paper seeks to rectify this situation.

### Anomalous scattering

In anomalous scattering, the atomic scattering factor *f* takes on a complex form due to absorption near an atomic absorption edge, and is energy- or wavelength (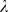)-dependent [3]: 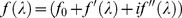
(1)


with magnitude
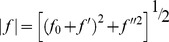
(2)


with 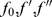 all real.

Away from an absorption edge, the additional terms 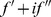
are small, so that the scattering factor reduces to the wavelength-independent, real factor 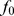.

### Previous biological applications of ASAXS

Stuhrmann [4] attempted to determine the relative locations of the four iron atoms in the haemoglobin molecule. By using absorption versus wavelength measurements Stuhrmann obtained the imaginary part, 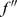, of the scattering factor of the bound iron atoms; the real part, 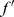 was deduced by the application of the Kramers-Kronig relationship. The scattered intensity at various wavelengths was then corrected for absorption using the derived 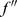 values. Relative intensity variation with wavelength was derived by subtraction of the corrected scattering curves from each other thus removing all the non-anomalous scattering. In the relative intensity data, the iron-iron scattering terms, which relate directly to the iron-iron distance distribution, were estimated to contribute only ∼10^−5^ of the scattered intensity for the molecule at zero angle, and were thus neglected in the analysis. Thus the remaining anomalous contribution results from the cross-terms i.e. iron-atom scattering, which depend on 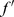. These cross-scattering intensity terms were fitted using intensity curves at 30 different wavelengths, to obtain good statistics for the term since even this component is only 10^−3^–10^−2^ of the total intensity. By use of multipole expansions for the scattering density, and assuming a simple density distribution for the atoms in the molecule, the distance distributions for the iron atoms were estimated. The distances derived were shown to be consistent with data, but it was not possible to be any more accurate than that regarding the position or distance between iron atoms since it relied on knowledge or estimation of the atomic structure. Das *et al* [8] used ASAXS to determine the extent of the ion cloud around DNA, although they were restricted to modelling DNA as a simple rod. However the study did show the potential of ASAXS to look at specific ion types within a biological system.

An improved method of ASAXS, and one that proposed using multiple labels, was derived in a theoretical study by Munro [9] using modulated or derivative analysis of the signal. The derived anomalous signal was claimed to be a factor of 10 better than standard anomalous scattering (for binary or ternary systems *i.e.* those with only 2 or 3 types of scatterer). Munro found that for 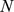
distinct scattering species there are 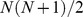 distinct partial structure factors. A simulation demonstrated the extraction of partial structure factors for binary and ternary systems, and generalised equations for 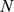
species were also given. Munro applied randomly generated errors to the intensity in the simulations, and showed how these errors propagate through the inverse matrix solution. Jemian *et al.* [10] also present modulated anomalous X-ray scattering data, showing an apparent improvement over standard ASAXS in the errors in obtaining the partial structure factors.

It is clear that earlier workers found the strength of the label-label anomalous scattering term in labelled molecules (rather than binary or ternary systems) to be difficult to extract from the overall scattered intensity. To increase the anomalous signal, Miake-Lye *et al*. [11] used terbium as label, as it has a high anomalous scattering effect (significantly greater than for iron). Using terbium at the calcium binding sites in parvalbumin, they attempted to determine label-label distances. However, their absorption correction for 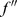 was carried out using the absorbance of TbCl_3_ solution rather than parvalbumin, and the authors believe that the resulting error made it impossible to extract the Tb-Tb interference term, which was of similar magnitude to that error. They were, however, able to describe the theoretical dependencies with wavelength of the 3 different component terms: atoms, label-atoms, label-label, and estimate the contribution of each. From their experimental data, like Stuhrmann [3]–[4], they were able to derive the cross-scattering term. In this case, the authors used a spherically symmetric Gaussian model for the scattering density of the molecule and placed the two terbium atoms at specific radii. Artefacts of the model meant that the fitting parameters adjusted such that the Tb always came out on the surface of the molecule, and the scattering was insensitive to the anomalous components. However, the major conclusion of this study was that the preferred technique is to obtain the interference for the Tb-Tb term, but that this requires very good subtraction of absorption and fluorescence, as well as an excellent account taken of the beam variation corrections.

A more recent study by Stuhrmann [12] made use of the technique of contrast variation to match the solvent as much as possible to the molecule scattering density. Hence any scattering is then due to the labels only. Stuhrmann also derives useful estimates of the change in intensity due to anomalous or resonant atoms in a molecule at low and high resolution.

The potential for the use of ASAXS to determine the relative positions of label atoms bound to a molecule was thus identified many years ago by workers such as Stuhrmann [4] and Miake-Lye et al. [11]. However, the measurements at that stage were not found to be sufficiently accurate to extract the label-label interference term which relates directly to the label distance distributions. Developments and improvements in instrumentation, beam stability and controllability, and detection suggest that it may now be possible to detect that interference term. Thus, anomalous scattering measurements could be used as a “molecular ruler” by the attachment of labelling atoms to sites on a biological macromolecule. Mathew-Fenn *et al*. [13]–[14] published two papers in which they report the use of standard SAXS on labelled DNA molecules of different lengths in order to measure the length of the helix. Lipfart and Doniach [15] also hypothesised how gold labels, appropriately positioned, could be used to determine distances across biological assemblies. Most recently Tainer and co-workers [16] used nanogold labels and non-anomalous SAXS to probe DNA conformations in solution. While successful, this was for a single metal type. We extend this approach theoretically to the anomalous case, and to multiple metal types, and investigate by simulation the current feasibility of using anomalous SAXS as a molecular ruler. By employing ASAXS, measurements will be able to be made using multiple wavelengths obtained on a single sample, thus removing sample-sample variation inherent in previous methodologies where unlabelled, singly and then doubly labelled samples had to be measured at a single wavelength and data subtracted.

### Theory

This section presents the theoretical background to the determination of the distance between label species on molecules. This determination assumes that measurements are made of scattered intensity as a function of scattering angle at a set of tuned beam energies (wavelengths).

### Molecule with labels

Consider a biomolecule in solution with *n* different types of heterogeneous atom or other label species attached. We can separate the contributions of the various wavelength-dependent terms to the total intensity as follows:
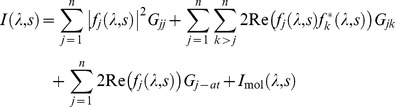
(3)


where 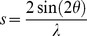



with 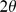 the scattering angle, and the subscripts *j,k* denote a label type not an individual label scatterer. The final term in the equation, 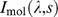, is the scattering intensity produced by the atoms of the molecule only (without labels or heteroatoms). The terms denoted 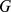 correspond to partial structure factors which are related to the distance probability distributions 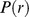 as follows:
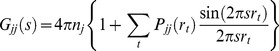
(4)

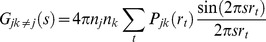
(5)

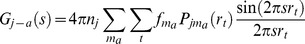
(6)


where the subscript *m_a_* refers to an individual atom. The probability distributions 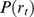 are the non-normalised probabilities of a label-label or label-atom pair being at a given (discretised) distance apart, 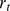. The parameters 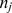 and 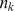 denote the numbers of labels of type *j* and *k* respectively. Note that where the term “atom” is used to identify a scattering term, this is taken to refer to that resulting from the atoms of the organic molecule, excluding any attached labels. Even where the labels are atomic, they are referred to as “labels” and are not included in the “atom” designation.

The first two terms in equation 3 relate to label-label scattering pairs, the first of these for labels of the same type, and the second for dissimilar labels. The third term in equation 3 relates to scattering between labels and the atoms of the molecule, and the final term is the scattering from pairs of atoms of the molecule. Each of these terms is related to a partial structure factor which are defined in equations 4–6 in terms of distance distribution functions. These distribution functions essentially define the number of pairs (of atoms, or labels or atom-label combinations) at any given distance apart. We have included the atomic scattering factor 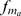 in the definition for the partial structure factor between labels and atoms of the molecule. Although this does not conform to the standard definition of a partial structure factor, it is done this way for convenience for the matrix inversion to obtain the distance distributions.

It is the different wavelength dependence of the various terms which permits their separation from the measured intensity data. The label-label interference terms have a wavelength dependence defined by the parameter 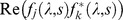, which for labels of the same type is equivalent to 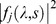. The cross-scattering terms between labels and the atoms of the molecules has a wavelength dependence dominated by the anomalous scattering of the label, and thus depends on the parameter 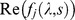 for each label type. Since most organic atoms have only a small anomalous contribution, their wavelength dependence has been neglected. The atom-atom scattering terms which comprise the final term in the intensity may also have some weak wavelength dependence. Although this is expected to be a small contribution, it can make a significant difference to the extracted label-label terms. As an estimate of the wavelength dependence we have used the mean squared *f* of the atoms in the molecule, 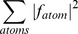, which is proportional to the self-scattering terms which dominate at small *s*. In total, for *n* different types of label, there are 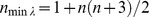 partial structure factors to be deduced, and therefore a minimum of 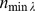 different wavelengths must be used to resolve them. This comprises 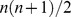 label-label pair terms, 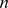
label-atom pair terms, and one term for the molecule scattering (atom-atom pairs).

For the purposes of this study, we are interested only in the interference terms between label species, 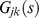 and the corresponding distance distributions for the labels 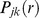.

### Matrix solution

This section demonstrates how the label-label partial structure factors can be obtained from the scattered intensity measurements, using a matrix inversion technique, derived from the equations presented above. In an ASAXS experiment, the beam energy is tuned to produce a beam of a certain wavelength, and then the scattered intensity from the sample is measured as a function of scattering angle (giving a set of different values for the scattering parameter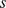). Measurements are made at 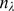 different wavelengths, and at each wavelength the scattered intensity is measured at 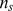 values of momentum transfer vector magnitude 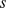. The intensity data 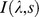 is therefore obtained in the form of a set of column vectors of intensity as a function of the momentum transfer vector *s*. The set of these vectors consists of measurements 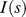 at each wavelength. However, in order to enable a single matrix solution, matrices are constructed to include all wavelengths and *s*-values. The intensity matrix is formed directly by concatenating the 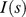 column vectors at the various wavelengths thus
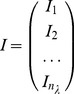
(7)


where each of the 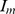 represents an 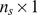 column vector which holds the intensity at each *s*-value at the wavelength 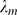 such that 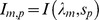 i.e. the intensity at wavelength 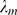 and the *p*'th scattering vector value. The subscript *p* refers to the element location (row number) in the matrix 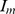. Thus, the overall intensity matrix is a column vector of dimension .

Similarly we can construct a matrix for the partial structure factors, including terms for label-label, label-atom and atom-atom structure factors, and with the *s*-dependence for each. The partial structure factors are independent of wavelength, since they represent a characteristic of the material itself. This matrix is the “unknown” which we aim to determine by inversion of the matrix equation. 
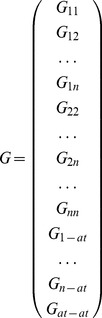
(8)


Here the label-label partial structure factor components 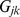 each represents a matrix (column vector) of dimension 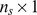 which define the values 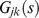 such that the matrix element *p* of 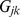 is 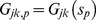
(9)


where 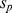 is the *p*'th momentum transfer vector value (see equation 4–5). The label-atom and atom-atom sub-matrices are similarly defined by 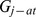 and 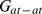 respectively, representing matrices of dimension 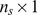 such that the matrix element *p* of each is
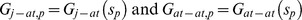



where 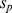 is the *p*'th scattering vector value (see equation 6).

Then the intensity and partial structure factors can be related through the matrix equation
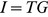
(10)


where the scattering matrix *T* is constructed from the scattering factors for each combination of label types and atoms (see equation 3) thus
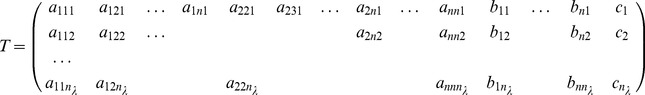
(11)


Each term in this scattering matrix (equation 11) represents a square diagonal matrix of dimensions 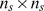 holding the appropriate value of the scattering factors at the corresponding wavelength and *s*-value.

For label-label type combinations, each term 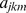 denotes a square matrix of dimensions 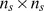 for label type *j* with label type *k* and for a wavelength 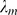 represented by subscript *m*. The element 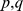 of the matrix 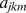 is therefore defined by
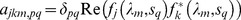
(12)


by comparison with equation 3, where *p* and *q* represent the row and column number, and 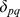 is the Kronecker delta such that
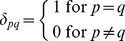
(13)


The label-atom scattering terms, 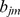 denote a square matrix of dimensions 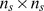 for scattering between label type *j* and the atoms of the molecule for a wavelength 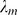. The elements of the matrix are defined by (compare with equation 3)
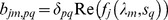
(14)


Similarly for the scattering between atoms of the molecule, the terms 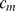 denote a square matrix of dimensions 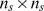 for the scattering contribution at wavelength 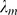. The matrix elements are
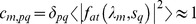
(15)


It should be noted that the order of the columns in the scattering matrix *T* corresponds to the order of the rows in the partial structure factor matrix *G*. This order is for the label types of all combinations *jk* with 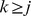 in the order *11,12,…1n,22,…2n,…nn*, followed by each label type *j* scattering with atoms (in the label type order *1…n*), with the atom scattering terms last of all.

The matrix is conditioned by dividing each element by the corresponding scattering matrix term (e.g. label type 1 with label type 2) at the first wavelength and the first scattering vector value. Thus 
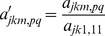
(16)

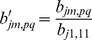
(17)

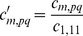
(18)


Inverting the matrix equation 10 leads to the determination of the partial structure factors thus:
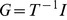
(19)


Since there are 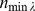 unknown structure factors, there must be a minimum of 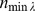 wavelengths at which intensity measurements are made. However, the error statistics can be improved by using more wavelengths, say 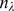 and taking a minimum least squares error approach to the solution. In the general case, with *s*-dependent scattering factors 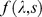 for the label species, the *T*-matrix is square and of size 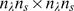 where 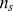 is the number of *s*-values at which measurements are made. The partial structure matrix *G* has 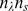 values (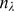 structure factors each of length 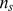) and the intensity vector is similarly composed of 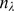 sets of 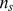 values. In the simpler case where the labels are atomic, so that their scattering factor is wavelength-dependent, but not *s*-dependent, 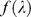, the *T* -matrix can be reduced to a square matrix of size 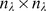. The inverted matrix can then be applied at each *s* to obtain the partial structure factors from the intensity 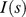.

As an example, consider a biomolecule labelled using two identical label atoms, with no 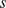-dependence of their scattering factors. Measurements of 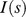 are made at three wavelengths to isolate the three partial structure factors. The matrix simplifies to 
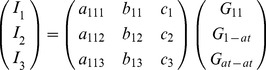
(20)


where the sub-matrices are defined by elements *p,q* such that
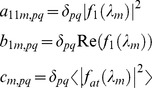
(21)


The inversion of equation 20 enables the partial structure factor to be determined for the label-label interference term in this example, which is related to the distance distribution for the label pairs by the following relationship
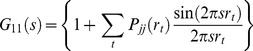
(22)


Similarly, for a more complex problem, with multiple label types, and with *s-*dependent scattering factors for the label species, the matrix equation 10 can be inverted to extract the partial structure factors for the label-label terms. From these, the distance distributions for the label pairs can be extracted.

### Distance distributions

Having demonstrated how the partial structure factors can be extracted from the scattered intensity measurements, it is now necessary to consider how to determine the distance distributions for the label pairs from them. The partial structure factors 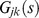 for the label-label interference terms (equations 4–5) look slightly different for pairs of labels of the same type and for dissimilar pairs. This is due to the self-scattering contribution for labels of the same type, corresponding to zero separation *r* = 0, where the sinc function is unity. This self-scattering contribution has been written separately from the distance distribution *P*. In the numerical calculation, the self-scattering term for similar labels is removed from the structure factor to retain only the sinc functional dependence (see below, Simulations section). Hence, equation 5 is valid for both similar and dissimilar label pairs. Theoretically, a direct inversion from the partial structure factor is available, given by
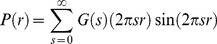
(23)


neglecting constant factors. However, this was found to give poor results, perhaps because the summation can only be carried out on a finite *s-*range in practice. Instead, a set of basis functions 
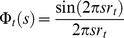
(24)


are defined for a discrete set of distances 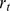 and the distance distribution for these discrete distances is fitted to the partial structure factor as follows
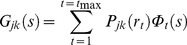
(25)


A similar approach was used by Mathew-Fenn *et al* [13].

In a rigid molecule, the label-label distance distributions define the relative position of the labels; for example *P(r)* may be a spike at a certain separation *r*. However, for molecules with flexible domains, the distribution may represent the relative likelihood of the separation between the labels, resulting from an ensemble average of molecules with many conformations. As such, it is expected that the maximum peak height will decrease with increasing flexibility, leading to an ultimate reduction in the maximum observable label/molecule signal. Practically for DNA this will mean a reduction in the overall length of the DNA molecules that can be analysed.

This Theory section has presented the methodology for extracting the distance distribution (or simply the distance apart) for label species attached to a molecule from scattered intensity measurements at a number of beam wavelengths and scattering angles. In order to assess the feasibility of the method, calculations have been carried out using simulated data for scattered intensity, and determining the distance distributions from that data, as if it were real experimental data. These simulations are presented in the next section.

## Simulations

The feasibility of the use of anomalous SAXS as a “molecular ruler” was investigated by simulating scattering intensity data for biomolecules with attached labels. These simulated data were then analysed, as if they were experimental measurements, using the methods detailed in the Theory section to obtain the distance distribution functions for the labels, and hence determine inter-label distances. Simulations were carried out using code developed in MATLAB version R2009a-R2012a (The Mathworks, Inc.) for a set of DNA molecules of varying lengths. Label species of gold atoms, gold nanocrystals, and platinum nanocrystals were used in the simulations. The code was run on a 3 GHz PC running Windows XP. The MATLAB codes are provided as supplementary materials in zipped file Programs S1.

The workflow of the process was as follows (A) Obtain the pdb file for the selected molecule. (B) Calculate the molecular scattering intensity in solvent. (C) Add label atoms into the pdb file at specified coordinates. (D) Calculate the label-biomolecule scattering and the label-label scattering using MATLAB program at specified X-ray energies (wavelengths). (E) Sum the intensity contributions to obtain the total scattered intensity as a function of the magnitude of the momentum transfer vector, *s* at each energy. (F) Add normally distributed random errors. (G) Construct the scattering matrix *T* using the wavelength-dependence of the label scattering factors. (H) Invert matrix to obtain label-label partial structure factors *G*. (I) Truncate and shift *G*(*s*) to reduce errors. (J) Obtain the distance distribution function *P*(*r*) by least square errors and maximum entropy techniques. Each of these steps is now considered in more detail.

### Coordinate files (A)

The pdb-format coordinate files for the set of DNA molecules were generated using the make-na server [17] which is based on the Nucleic Acid Builder code produced by Case and others [18]. The sequence provided to the make-na server was specified for only one strand, so that a blunt-ended Watson-Crick helix is produced. Calculations were carried out for 10, 20, 50, 100 and 200 base pair DNA, using B-type helices. The sequence for the 10-base pair DNA was identical to that used by Mathew-Fenn *et al*. for the ‘A’ strand [13]; other DNA sequences were produced using a random number generator to select the sequence. Table 1 shows the sequences for the DNA duplexes used in the calculations.

**Table 1:** DNA Duplex sequences.

Number of base pairs	Sequence
10	GCATCTGGGC
20	ACTAAAGGGCGCGAGACGTA
50	ATATTTACCTCTACAATGGATGCGCAAAAACATTCCCTCATCACAATTGA
100	GATTGTGCGAGACAATGCTACCTTACCGGTCGGAACTCGATCGGTTGAACTCTATCACGCCTGGTCTTCGAAGTTAGCACATCGAGCGGGCAATATGTAC
200	AGCGCTGCTACCGGTTCATGTGGTAACGAACTCGCGTATTCAATCGACGGAGAGGTGCATCCTGGTCTCAATGCGATTGTGCCCTCTTTCGCCAGGATGCGTCCTTGAGGGGCTTGGTGCATCTCCACTCCTGATACAAGTGGACCATTAGGAAGATTTGGCAACTTCCACCGGATAAAGAAACGGCTTCGTTTTTACTT

*The DNA duplex sequences used to produce the pdb files.*

### Scattering intensity in solvent (B)

In real measurements, the biomolecules exist in a solvent, usually water or a buffer solution, and the scattering which would have been received from the volume of solvent now occupied by the biomolecules must be subtracted from the total scattered intensity. In addition, biomolecules in solution have a hydration layer, and the scattering density of this layer will be different from that of the bulk solvent; this effect must also be accounted for. Both terms are discussed extensively for the calculation of scattering intensity from biomolecules in solution by Svergun and co-workers [19]. In order to account for solvent and hydration layer effects, the molecular scattering intensity in solvent was calculated using CRYSOL [19] using the coordinate pdb file with no label atoms or nanocrystals added. Hydrogen atoms were excluded from the calculation, and default values were used for the solvent properties (solvent density 0.334 and hydration shell contrast 0.03) and calculation parameters (maximum order of harmonics, 15, order of Fibonacci grid, 17). Results were calculated up to 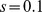 Å^−1^ with 201 points. The intensity results were later linearly interpolated in the MATLAB code to obtain a finer discretisation in *s*. It should be noted that the atomic scattering factors used by CRYSOL are wavelength-independent. Thus, for each molecule, a single scattering intensity curve is obtained, which is taken as the scattering intensity from the molecule in solvent at all energies.

In the Theory section, however, it was shown that the extraction of the interference scattering term between attached labels depends only on the wavelength dependence of the various contributions. Since the solvent contrast and hydration layer terms are wavelength independent, as is the scattering from the biomolecule in vacuum, these two terms act only as a shift on the biomolecule scattering contributions, and do not affect the determination of the label-label contributions. However, the solvent effects were included in the simulations in order to reproduce experimental conditions as closely as possible.

### Attachment of label atoms or nanocrystals (C)

Both atomic and nanocrystal gold label species were tested in the calculations. The nanocrystals were defined to be of radius 7 Å and incorporating 78 gold atoms each; these parameters were the same as those of Mathew-Fenn *et al*. [13]. In addition, a platinum nanocrystal was simulated, which was taken to have the same radius and number of atoms as the gold nanocrystals. To our knowledge, platinum nanocrystals are only currently available in the nanometre size range.

To test the use of atomic labels, the pdb file of the 10 base-pair DNA was modified by the addition of a single gold atom at the 3′ end of each strand. The atom was located at a distance of 1.48 Å (an oxygen-oxygen bond length) from the terminal oxygen atom, along the direction of the outermost oxygen-hydrogen bond. The hydrogen atom was removed.

**Figure 1:**
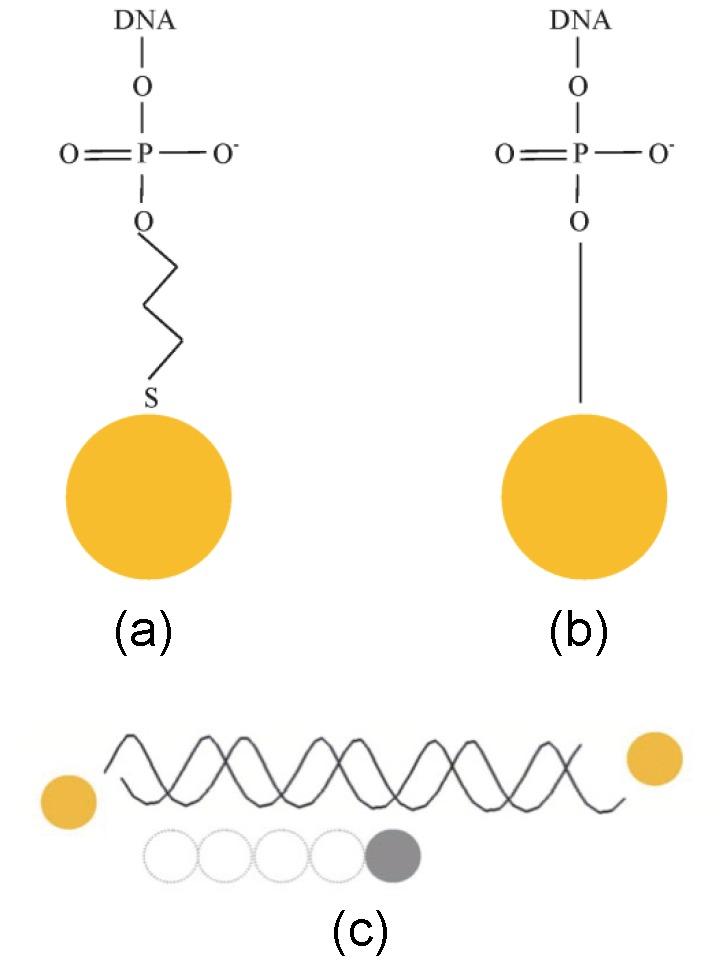
Diagram of nanocrystal attachment to DNA molecule (a) the thiol linkage to the gold nanocrystal [13] (b) gold nanocrystal position as defined in coordinate file (c) gold and platinum nanocrystals on a 50 base-pair DNA molecule. The dotted circles show the various positions for the platinum nanocrystal.

Attachment of gold nanocrystals to DNA molecules was achieved by Mathew-Fenn *et al*. [13] by the use of thiol-modification of the DNA. The *process* of attachment is not the focus of the present study; we have, however, determined the location of the nanocrystals based on the gold-thiol structure given by those workers, see Figure 1a. The sulf-hydryl group of the thiol-modified DNA attaches directly to the gold nanocrystal. Between the oxygen atom on the DNA molecule (shown) and the gold nanocrystal, there are four bond linkages (C-O, C-C and C-S). Taking these to be at a 45° angle, and all the same length of 1.480 Å, the distance from the oxygen atom to the centre of the nanocrystal was taken to be 2√2 times the bond length plus the radius of the nanocrystal, that is 11.186 Å. The oxygen-nanocrystal direction was in line with the phosphorous-oxygen bond; the intervening linkage atoms were not included (Figure 1b). In each DNA molecule coordinate file, a gold nanocrystal position was defined at the 3′ end of each strand. The distance between the centre of the nanocrystals in the various molecules is shown in Table 2. These distances do not follow a regular scaling with the number of base pairs for the DNA molecules; this is because the nanocrystals are positioned off the helix axis by a significant distance, and this affects the inter-nanocrystal distances disproportionately for the smaller molecules.

**Table 2:** Distances between label atoms or nanocrystals.

Molecule	Actual distance between labels/Å	Calculated distance between labels/Å
10 bp DNA, atom labels	37.3	----
10 bp DNA, nanocrystal	50.5	51
20 bp DNA, nanocrystal	60.7	61
50 bp DNA, nanocrystal	142.0	143
100 bp DNA, nanocrystal	269.6	270
200 bp DNA, nanocrystal	672.0	673

*The distance between the label atoms or nanocrystals, as defined in the coordinate files, and determined by the anomalous SAXS simulation.*

For the purpose of demonstrating the molecular ruler method using nanocrystals of different types, simulations were carried out using a platinum nanocrystal ‘attached’ to the 50 base-pair DNA molecule, with gold nanocrystals at each end. The platinum nanocrystal was located arbitrarily at locations offset from the helix, and at varying positions along the axial direction, to simulate a range of gold-platinum distances (see Figure 1c). The distances between the coordinates of the nanocrystals are given in Table 3.

**Table 3:** Distances between nanocrystal labels.

Label	Actual distance Au-Au/Å	Calculated distance Au-Au/Å	Actual distances Au-Pt/Å		Calculated distances Au-Pt/Å	
(i)	142	144	74	-	74	-
(ii)	142	145	60	90	61	89
(iii)	142	146	50	100	52	97
(iv)	142	145	40	112	41	111
(v)	142	143	30	127	32	129

*Actual and calculated distances between gold and platinum nanocrystals for a 50 base-pair DNA molecule, with a gold nanocrystal at each end, and a platinum nanocrystal placed at a variety of distances from each end.*

### Calculation of label scattering contributions (D–E)

Having obtained the scattering intensity for the molecule in solvent using CRYSOL, the contributions to the scattering intensity due to the label-atom scattering and label-label interference terms were then added using the MATLAB code. The contribution from label-solvent interactions is omitted. Simulation of the scattered intensity for the label-atom and label-label terms was based on equation (3) using a set of basis functions of the form 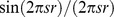
for a set of distances *r* between (and including) zero and a maximum value (determined by the size of the molecule), spaced at 1 Å intervals, and using a range 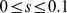 Å^−1^ with an interval of 10^−4^ Å^−1^ (1001 values of 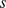). Typically around 1000 *s*-values can be obtained in an experimental measurement. The scattering factors for the labels are considered in the next section. The X-ray energies used in the calculations were based on the available Diamond X-ray source which is tunable between 11.6–12.4 keV. For calculations with a single label type (gold atoms or gold nanocrystals), five different energy values were adopted in this range, at an interval of 200 eV (set A, Table 3); a minimum of three energies is required in order to separate the label scattering contribution. Where both gold and platinum nanocrystals were simulated, nine energies were used at 100 eV spacing over the same range (set C, Table 3); a minimum of six energies is required when two label species are used. In fact, the beam energy at Diamond can be tuned to 1–3 eV resolution, which is considerably finer than the energy intervals used in most of the simulations reported here. Tuning the beam energy more closely to the absorption edge should result in greater differentiation of the label-label contribution from the molecule scattering intensity, and compensate for higher error levels on the intensity measurements. One set of calculations has been carried out for 50 bp DNA with gold nanocrystal labels with beam energies at 11.800, 11.912, 11.914, 11.916, 11.918, 11.920, 11.922, 12.000, 12.200 keV, close to the absorption edge (set B, Table 3). Since the scattering from the label species are both wavelength (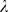)- (energy) and wavevector (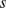)-dependent, the scattering intensity was calculated for each X-ray energy, for the set of 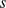-values. These terms were added to the scattering intensity of the molecule in solvent (see previous section) to obtain the simulated scattering intensity for a labelled molecule in solvent.

The scattering factors for the atoms of the molecules, and for the label atoms were obtained from the Lawrence Berkeley X-ray data site [20] and interpolated to the required energies. These were used to calculate the label-atom and label-label scattering intensity contribution.

If the label species are not atomic, but are nano-crystals or other scatterers of significant size, the scattering from the label itself has dependence on the magnitude of the momentum transfer vector *s*. This is due to the self-scattering term from interference between the scattered field from different parts of the label. The effect can be incorporated into the scattering factor of the label, using the known result for the additional scattering factor for a sphere of uniform electron density:
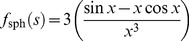
(26)


where 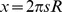 and *R* is the radius of the sphere [21]–[22]. This was the form used by Mathew-Fenn et al. in their work on DNA labelled with gold nanocrystals [13]. For a spherical label containing
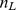
atoms with atomic scattering factor 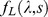, the scattering factor for the label becomes
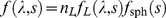
(27)


This was the form used for the scattering factors for the nanocrystal label species, with the appropriate nanocrystal radius (7 Å) and number of atoms (78).

### Addition of pseudo-random errors (F)

Random experimental errors were simulated by adding normally-distributed pseudo-random values to the intensity, with a standard deviation proportional to the total intensity at zero angle 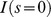. Typical experimental errors are in the range 0.01–0.1% of 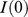. For most of the calculations presented here, errors were taken to be at the lowest limit of 0.01% of 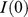 (sets A and C). This is a low level of error, requiring highly accurate measurements, but will permit the feasibility of the technique to be explored. One set of calculations (set B, for 50 bp DNA with gold nanocrystals and using 9 different beam energies) was carried out with errors at the higher level of 0.1% 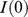 to explore the limits of the technique. The effects of the level of experimental error will be considered later.

### Construction of T-matrix (G)

The scattering matrix *T* is constructed using the scattering factors of the various species, labels and atoms, based on equation 11. To account for the small effect due to the wavelength-dependence of the molecular scattering contribution, a mean scattering factor was used, averaged over all atoms in the molecule to obtain the matrix components for atom-atom scattering. Although in some experimental situations, the full chemical sequence of the molecule may not be known, some estimate of the mean scattering factor could be applied. For atomic label species, the scattering factors were taken to be independent of the magnitude of the momentum transfer vector (*s*), resulting in the simplified form of the equation (20). For nanocrystal labels, the full *s*-dependence of the scattering factors (see previous section) were incorporated into the scattering matrix.

### Inversion of matrix to obtain partial structure factors (H)

The matrix inversion according to equation 19 was achieved using the matrix left division function in MATLAB, which selects an appropriate inversion algorithm dependent on the character of the matrix *T*. In the case where intensity data is available at a greater number of energies (wavelengths) than the minimum required, 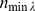, to separate all contributions from the scattering pairs, the system is over-determined. Then a least-squares solution is determined by the MATLAB function, which finds 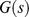 that minimises the 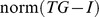.

### Correction of the 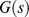 partial structure factors (I)

The contribution of the label-label terms to the overall scattered intensity can be small when the labels are atoms rather than nanocrystals, or for large molecules. This problem led to the difficulties experienced by earlier workers who were unable to isolate the label-label contribution, as reported in an earlier section. Experimental errors in the intensity measurements, and the small inaccuracies in the assumptions of the analysis (such as the *s-*independence of the atom scattering) can lead to errors in the label-label partial structure factors which are very large. In order to improve the accuracy of the analysis, two techniques were applied to the label-label partial structure factors before the distance distributions were calculated. These were truncation and removal of the self-scattering component.

Firstly the 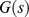 data for the label-label pairs was truncated at a maximum value of *s* at which the errors exceeded an acceptable level. This was determined visually from a plot of 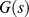, using smoothing to assist the identification of the point at which the signal to noise ratio becomes unacceptably high. An example of a plot of an extracted label-label 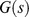 is shown in Figure S1, obtained using a simulated error level (on intensity) at 0.01% 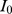. Truncation was chosen to be where the oscillatory nature of the function can no longer be distinguished through the random errors. The truncation was therefore different for each calculation; the truncation limits are given in Table 4.

**Table 4:** Parameters for simulations.

Molecule	(a) 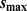 /Å^−1^	(b) 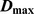 /Å	(c) 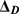 /Å
**Set A**:			
10 bp DNA, gold atom labels	0.1	60	1
10 bp DNA, gold nanocrystal	0.08	60	1
20 bp DNA, gold nanocrystal	0.075	70	1
50 bp DNA, gold nanocrystal	0.04	180	2
100 bp DNA, gold nanocrystal	0.05	350	5
200 bp DNA, gold nanocrystal	0.04	700	5
**Set B**:			
50 bp DNA, gold nanocrystal	0.05	180	2
**Set C**:			
50 bp DNA, gold/platinum nanocrystal	0.04	180	2

*The parameters used in the calculations for each molecule type. (a) the* 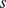 *value at which the* 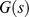 *function is truncated before inversion to obtain distance distributions. (b) the maximum inter-particle separation used for basis functions for inversion of* 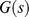 *(c) the spacing between basis functions.*

*Set A: errors at 0.01% I(0) and energies of 11.6–12.4 keV at 200 eV intervals. Gold atom or nanocrystal labels.*

*Set B: errors at 0.1% I(0) and energies of 11.800, 11.912, 11.914, 11.916, 11.918, 11.920, 11.922, 12.000, 12.200 keV. Gold nanocrystal labels.*

*Set C: errors at 0.01% I(0) and energies of 11.6–12.4 eV at 100 eV intervals. Gold and platinum nanocrystal labels.*

Secondly, the data were shifted so that the sinusoidal oscillations in 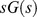
occur about a mean level of zero. This removes the self-scattering term in the partial structure factor for label pairs of the same type, and corrects any mean value errors for dissimilar label pairs. The procedure is similar to that described by Mathew-Fenn *et al* [13]. We find the offset value 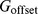 to minimize the parameter 
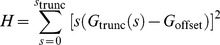
(28)


The resulting truncated and shifted partial structure factor 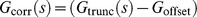 is then used in the inversion to obtain the distance distribution function.

### Inversion to obtain distance distributions (J)

The partial structure factors 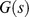 obtained for each label-label pair were then inverted to obtain the distance distribution, 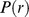, according to equations 24–25 using a least-squares non-negative optimisation. In regions of *s* where the shape factor for the label becomes very small or zero, errors in the inversion to obtain the structure factor 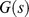 are amplified, so that very large errors occur in 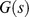 in these regions. This problem can occur near the zeros of the shape factor, and at large values of *s* where the shape factor and 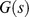 also become small. In order to reduce the impact of this effect, the fitting of 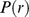 to the partial structure factor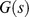 was weighted using the square of the sphere shape factor for the appropriate label species. The use of 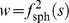
as a weighting causes the fit of 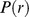 to be weighted to the best data, reducing the contribution of the most error-prone regions. The least squares constrained optimizer therefore finds 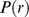 that minimises the 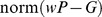. This was found to improve the accuracy of 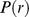 significantly. For atomic labels, no such weighting is required.

Further improvement in the distance distribution function is obtained by use of a maximum entropy optimizer. Following the procedure summarised by Mathew-Fenn *et al*. [13], the entropy is maximised with a regularisation constraint equivalent to the sum squared errors in 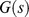. A control subset of the 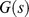 data is selected, consisting of 10% of the complete dataset, selected at random in five sections (to ensure coverage of the full range of *s*-values). The remaining data is used to obtain a distance distribution using the maximum entropy method, with the regularisation parameter chosen by annealing from a large value until the minimum least squares error in 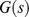 is reached for the control subset. This process (selecting a control subset, then finding the best regularisation parameter) is repeated 5 times, and the geometric mean of the regularisation parameters is obtained. This is then taken as the stopping value for the annealing of the regularisation parameter for the maximum entropy fit for the distance distribution on the full dataset. The initial solution for the maximum entropy calculation was taken as the constrained, weighted, least squares fit for 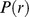.

The set of basis functions (equation 24) for the distance distribution fit were constructed using distances between zero and a maximum value, 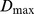, using 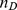
+1 values, giving a spacing of 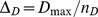. The values of these parameters for each calculation are shown in Table 4.

## Results

### Atomic labels

**Figure 2:**
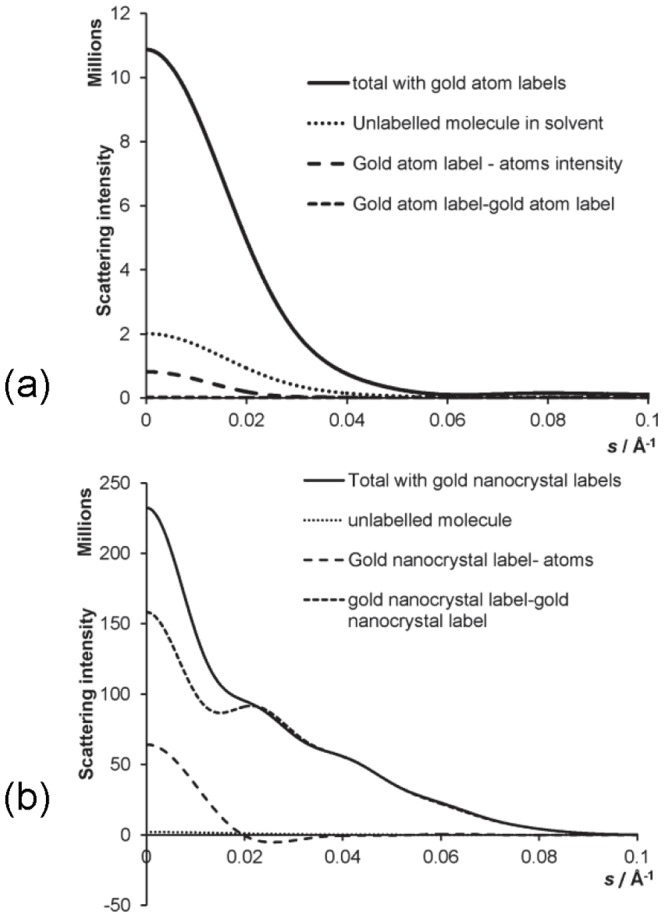
The intensity as a function of the momentum transfer vector magnitude, *s*, for a 10 base-pair DNA molecule in solvent at 12 keV beam energy showing the contributions from the molecule, label-atoms and label-label scattering (a) gold atom labels (b) gold nanocrystal labels. The total intensity includes simulated random errors (noise) but it cannot be seen on the scale of this plot.

Figure 2A shows the relative contribution to the scattering intensity of the molecule, label-atom and label-label scattering for 10 base-pair DNA with gold atomic labels attached, at 12 keV beam energy. The simulated random errors (noise) on the total intensity are included, but cannot be seen at the scale of this plot. The label-label contribution is only 0.2% of the total intensity at zero angle. The 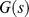 label-label partial structure factor which was isolated was dominated by noise, and the resulting inter-label distance distribution showed a number of peaks, none of which was related to the actual distance between the gold label atoms. Thus, even for such a relatively small molecule, it was not possible to obtain the distance distribution for *atomic* gold labels. Hence, further work focussed on the possible use of nanocrystals labels which have a much stronger scattering signature.

### Nanocrystal labels of a single type

The use of nanocrystal labels increases the contribution of the label-label terms to scattering intensity, thus allowing it to be separated from the measured intensity, hence permitting the determination of the label-label distance distribution. Figure 2B shows the intensity contributions for a 10 base-pair DNA molecule with gold nanocrystals. In this case the label-label scattering dominates the total intensity, being 68% of it at zero angle. As the number of base pairs increases, the label-label contribution becomes a smaller and smaller proportion of the total intensity, until eventually it can no longer be isolated to obtain distance distributions. Note that the error was taken as 0.01%*I*(0) for these calculations, and these are included in Figure 2B but are not visible at this scale. Further investigation of the effect of molecule size is presented in the Discussion section.

An example of the partial structure factor which was obtained for the nanocrystal-nanocrystal scattering for 50 base-pair DNA is shown in Figure S1 (see supplementary materials), before the baseline shift and truncation is applied. It is clear that the contribution of the random errors increases as *s* increases, hence the need for truncation before attempting to calculate the distance distribution. The oscillatory structure (resulting from the 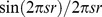 function pair-scattering dependence) can be identified up to ∼0.04 Å^−1^ in this case. For larger molecules (e.g. 200 base pair DNA), the simulated experimental errors make up a greater proportion of 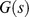 and it can be difficult to observe any structure in the data. Use of some smoothing on the plot helps to identify the oscillatory nature of the curve, and to decide on the truncation point; however, this smoothing was not included in the data used to obtain the distance distributions because it would violate assumptions in the least squares solver about the nature of the errors. The truncation points for each simulation are given in Table 4. A systematic investigation of the optimum truncation limit has not been conducted in the present study.

**Figure 3:**
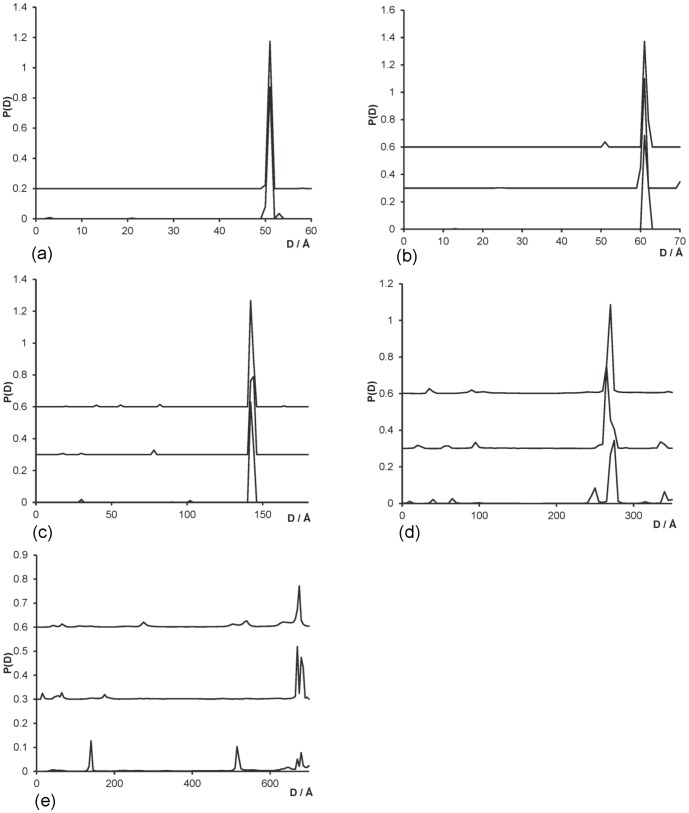
Results for label-label distance distribution *P*(*D*) of gold nanocrystals. (a) 10 bp DNA (b) 20 bp DNA (c) 50 bp DNA (d) 100 bp DNA (e) 200 bp DNA. Consecutive result sets are independent simulation runs; these are shifted vertically for clarity. The results shown are for least squares, non negative fitting for (a)–(c) and for maximum entropy fit for (d)–(e). Errors were simulated at 0.01% *I*(0).

Figure 3 shows a set of plots of the gold nanocrystal distance distributions obtained for the DNA molecules of various lengths (using errors of 0.01% *I*(0) and 5 beam energies at 200 eV intervals, set A). In each case, several independent simulations are shown, with the results shifted vertically for clarity; these sets were generated from the same intensity data, but with a different set of pseudo-random errors added. The mean of the distance between the nanocrystals at the peak of the distribution is shown for each of the molecules in Table 2. The inter-nanocrystal distance calculated from the results are accurate to within an Angstrom of the actual distance according to the coordinate definitions. Our calculations permitted determination of distance distributions up to and including 200 base pair DNA, where the label-label contribution to intensity is only 2.8% of the total at zero angle. A trial calculation using 500 base-pair was unsuccessful.

**Figure 4:**
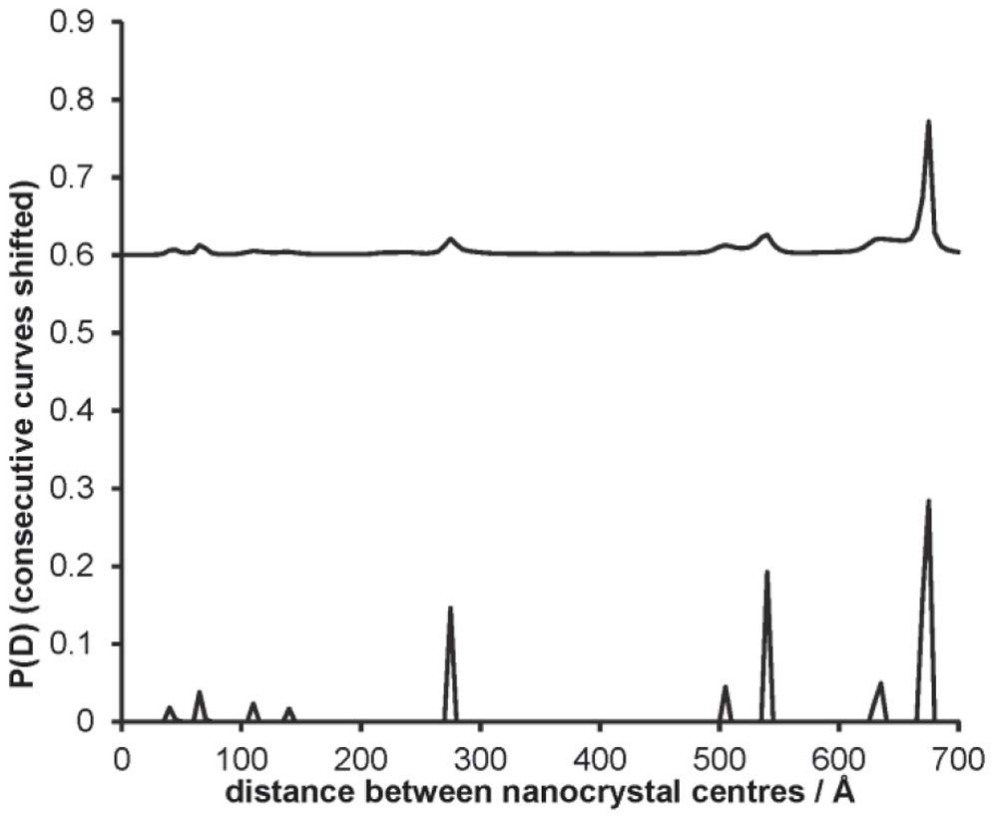
Comparison between least squares non negative fit for distance distribution with the maximum entropy annealed result, for 200 base pair DNA with two gold nanocrystals. The maximum entropy result has been shifted upwards by 0.3 for clarity.

It was found that the least squares fit for the distance distributions worked well for the smaller molecules; these fits are shown in Figure 3a–c. In these cases, the maximum entropy calculation often worsened the fit, by smoothing the sharp peak. However, for the larger molecules, where the nanocrystal scattering contribution is smaller compared with the background molecular scattering, the maximum entropy fit did improve the distance distributions which were obtained. Results for the 200 base-pair DNA had several subsidiary peaks in the least squares distance distributions, demonstrating that the technique is on the edge of its ability to discriminate the label-label scattering at this molecule size and error level. However, by using several data sets and the maximum entropy solver, it was still possible to obtain clear results for the distance distribution in that case. An example is shown in Figure 4 for 200 base-pair DNA. The maximum entropy calculation helps to reduce the amplitude of the spurious secondary peaks.

It should be noted that the width of the peaks in the distance distribution is influenced by the discretisation of distance which is selected for the basis functions (equation 24) i.e. by the values of *D*_max_ and *n_D_*. So, for example, with a 5 Å interval for the basis function distance values, the peak may be a single data point at a given inter-label distance, but the plotted line to the next data point (with zero probability) suggests a wider peak than that obtained if only a 2 Å interval were used. In fact, the width is representing only the uncertainty in distance due to the discretisation, if there is zero probability each side of the peak. In some cases, the method does not produce a sharp peak, for example Figure 3d for 100 bp DNA has in some simulations produced a broader peak due to the effects of noise on the uncertainty in the determination of the inter-label distance. In the case of flexible molecules, however, the distance distribution could be broad, representing the probability of the label locations being at a certain separation, taken as an ensemble average over all molecule configurations. Such information would provide valuable insight into the configurational behaviour of such molecules.

**Figure 5:**
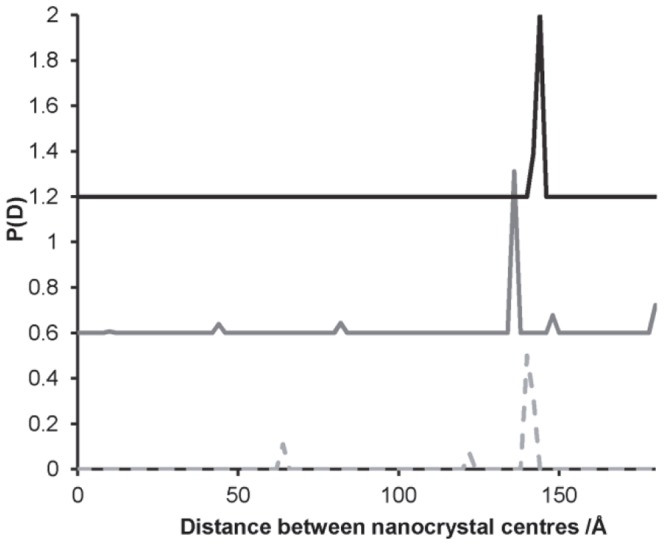
Probability distribution for the distance between nanocrystal centres for 50 base-pair DNA with gold nanocrystals at each end. Consecutive result sets are independent simulation runs; these are shifted vertically for clarity. Simulated errors in intensity were at 0.1% *I*(0) (set B) and the results shown are from the least squares solution.

The effect of larger errors on the intensity measurements was investigated by a set of simulations of the scattering from a 50 bp DNA molecule labelled with gold nanocrystals, with errors at the higher level of 0.1% *I*(0). To compensate for the increased error level, a greater number of different beam energies were used (i.e. different wavelengths) and these were selected close to the absorption edge of gold. The selected energies were 11.800, 11.912, 11.914, 11.916, 11.918, 11.920, 11.922, 12.000, 12.200 keV. The distance distributions (obtained from the least squares fit) are shown in Figure 5. Here there is some uncertainty in the distance between the nanocrystals, with each simulation producing a peak at a slightly different separation, although the *average* position of the peaks is at 140 Å (the actual separation is 142 Å). The maximum entropy calculation causes a significant broadening of the peak in some cases, and in others splits the peak into two separate peaks. It would appear that the technique is near the limit of its ability to extract the nanocrystal distances in this case.

### Nanocrystal labels of multiple types

**Figure 6:**
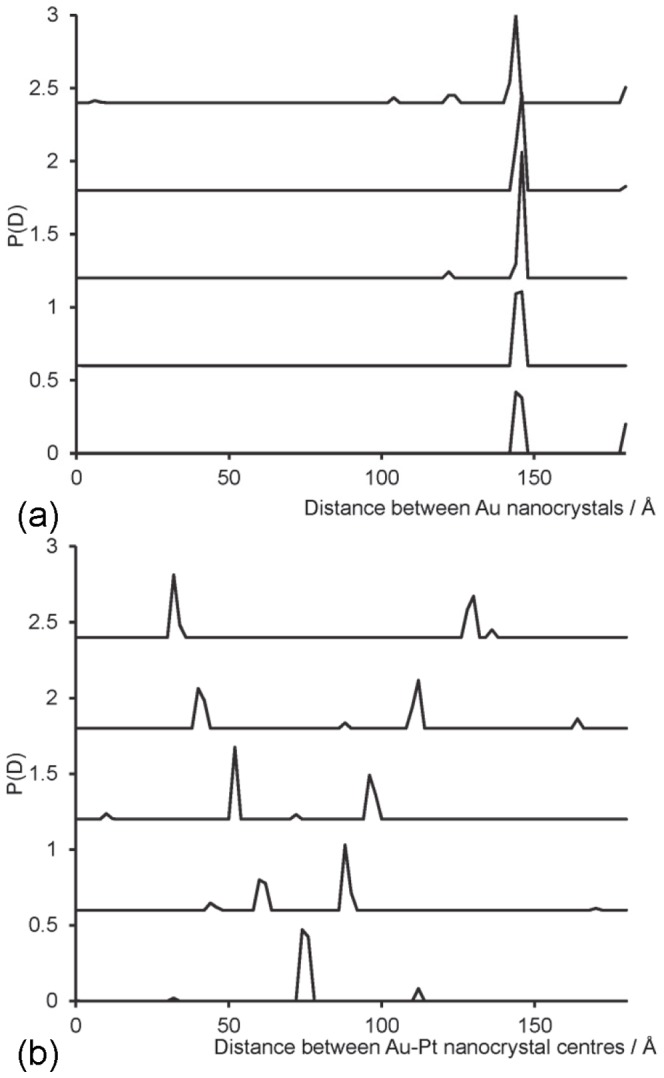
Probability distribution for the distance between nanocrystal centres for 50 base-pair DNA with gold nanocrystals at each end, and a platinum nanocrystal at some position between the ends. (a) Distribution for the gold-gold distance (b) distribution for the gold-platinum distance. In each case, each set of data is plotted shifted by 0.6 for clarity. The spacing between the Au-Pt nanocrystal coordinates are given in Table 3, and the curves are plotted for spacings (i)–(v) from bottom curve to top curve. Simulated errors in intensity were at 0.01% *I*(0) (set C).

Figure 6 shows the probability distributions for the distances between nanocrystals of gold and platinum on a 50 base-pair DNA molecule. The gold nanocrystals were positioned at the ends of the molecule, 142 Å apart, with the platinum nanocrystal placed at some position at varying distances from each end, firstly equidistant from the two gold nanocrystals, and gradually closer to one end on subsequent calculations. The distances between the coordinate positions are shown in Table 3. Calculations were carried out using a 2 Å spacing in the base functions, using parameters given in Table 4, and only a single simulation is shown for each platinum nanocrystal position. The calculated distribution for the gold-gold nanocrystal distance (Figure 6) shows a single sharp peak in the range 143–146 Å, indicating a slight reduction in accuracy compared with the case when only gold nanocrystals were used (Table 2), but still reasonably accurate. For the gold-platinum distance distributions, a single dominant peak can be seen for the case where the platinum is equidistant from the two gold nanocrystals, at 74 Å spacing, (Figure 6b (i)) an accurate measure of their separation. As the platinum nanocrystal is placed at different positions along the DNA; curves (ii)–(v) in Figure 6b; two peaks are seen with ever-widening separations, as the two distances to the respective gold nanocrystals become more distinct from one another. The positions of the peaks are given in Table 3, illustrating that the distances have again been determined to a high degree of accuracy. The resolution is only 2 Å (the spacing between the basis functions), but where the peak has significant probability across two points, the average distance was taken.

The results shown in Figure 6 are the non-negative least squares fits to the data. For the Au-Au distances distributions, a single sharp peak is observed, and for the Au-Pt two dominant peaks are seen, but a number of small spurious peaks also appear. Although the maximum entropy process successfully removed these secondary peaks for the Au-Pt distributions, it annealed too far for the Au-Au distributions, resulting in a poor quality result. Further tuning is required to optimise the maximum entropy calculation. It is believed that using more data points in 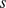
would improve the fitting, since the maximum entropy annealing parameter is determined by using subsets of the data, which only have a few data points unless the spacing in *s* is very small. The simulations with both nanocrystal types were carried out using 9 different beam energies (at 100 eV intervals from 11.6–12.4 keV, with errors at 0.01% *I(0)*, set C). A minimum of six energies is required to separate the various label and atom contributions to the scattering intensity with two label types, but simulations using only six energies were unable to resolve the distance distributions for either Au-Au or Au-Pt distances. Using a greater number of energies improves the data significantly, although limited computer memory constrained our simulations to 9 beam energies.

## Discussion

The results of the simulations presented in this study demonstrate that anomalous SAXS can in principle be used as a molecular ruler to measure distances in biological macromolecules by using metal nanocrystal labels. The criterion for success is determined by a number of factors; (a) the difference in the label-label scattering intensity at the different wavelengths – it is this difference which is used to isolate the label contributions, (b) the intensity scattered by the unlabelled molecule and (c) the magnitude of the experimental random errors. The intensity scattered by the unlabelled molecule is approximately proportional to the product of the square of the number of atoms and a mean squared scattering factor (the root mean square scattering factor was found to be around 7.5 for the DNA molecules). The difference in the label-label intensity between the various wavelengths, expressed by the change in the square of the magnitude of the scattering factor of the nanocrystal, can be denoted by 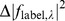. For a single gold atom, the magnitude of the scattering factor varies by ∼14 units over the energy range considered (although the simulations did not operate at the strongly varying absorption edge); for nanocrystals this must be scaled by the number of atoms in the nanocrystal, namely 78 atoms in our study. Thus the ratio of the label-label variation between wavelengths, to the background molecular scattering is given by 
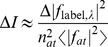
(29)


**Figure 7:**
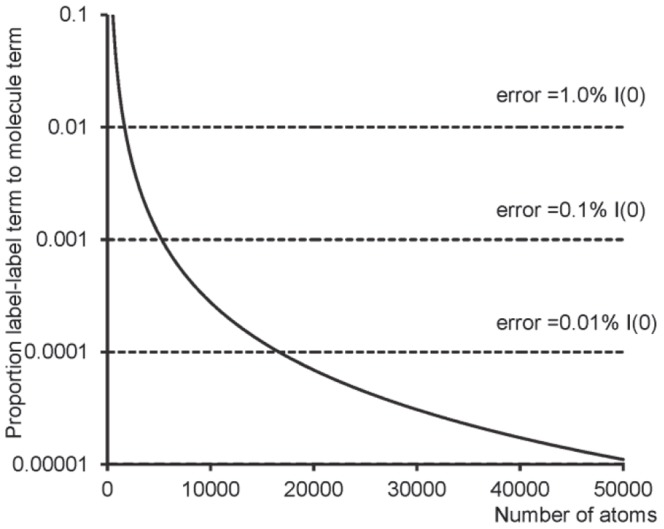
The ratio of the difference in the scattered label-label contribution to the molecular scattering intensity as a function of the number of atoms in the molecule (solid line), for gold nanocrystal labels. Also shown are the errors at selected levels (dashed lines). Intersection of the error line with the solid curve shows the maximum number of atoms in the molecule which can be used for that error level.

**Figure 7** shows how this parameter varies with the number of atoms in a molecule. As expected, as the molecule increases in size, the difference between the label contribution at different wavelengths becomes a smaller and smaller proportion of the total scattering intensity. When this ratio falls below the noise level caused by experimental errors, the label-label contribution will no longer be separable from the scattered intensity. A set of values for experimental errors (as a proportion of the total intensity) is also shown in Figure 7 (horizontal lines). The maximum size of molecule which can be used when the errors are at a specified level, can be obtained by the intersection of the curves. In the simulations reported here, successful determination of inter-label distances was achieved for 200 base-pair DNA (with 8194 atoms), but not for 500 base-pair DNA (20,494 atoms), with a simulated experimental error of 0.01% of 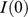. This is slightly better than might be expected from our estimate (equation 29). At the higher error level of 0.1% 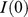 the nanocrystal separation was determined successfully (with some degree of uncertainty) for 50 bp DNA (2044 atoms), and the estimated maximum size of molecule at this error level (see Figure 7) is 5200 atoms. Thus, the formula given in equation 29 provides a useful guideline as to the likely success (or otherwise) of the technique for a given molecule/nanocrystal combination. For other nanocrystal types, the plot can be scaled by the appropriate number of atoms in the nanocrystal and the relevant scattering factor variation. It can also be used to judge whether experiments conducted with higher statistical error are feasible, and over what range of molecule sizes. For example, with an error of 1%
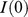
molecules up to only 1456 atoms would enable the label-label term to be distinguished. These estimates show that even with nanocrystal labels, highly accurate measurements are required to permit ASAXS distance measurements. This method provides a guideline to determine whether anomalous SAXS measurements are likely to be successful as a molecular ruler for a particular molecule and nanocrystal label.

The success of the technique can also be improved by tuning the beam energies close to the energy edge of the nanocrystal. Selecting energies at carefully chosen intervals clustered around the region where the nanocrystal scattering factor varies most strongly would improve the distinguishability of the label-label scattering contribution. This was demonstrated by the simulation for 50 bp DNA with 9 beam energies closely tuned to the absorption edge. However, there remains uncertainty in the scattering factor for the nanocrystals at beam energies close to the absorption edge, since the Berkeley data is only provided at much larger energy intervals, and the scattering factor of the nanocrystal may vary from the pure atomic value due to its attachment to the molecule. An experimental measurement of the absorption due to the nanocrystals at the required beam energies would be necessary to use very finely tuned beam energies.

One aspect that finally needs to be considered is flexibility. As DNA gets longer, it will start to act less as a stiff rod and exhibit more wormlike chain behaviour. Additionally, the linkers modelled in this study will themselves have an innate flexibility which will create uncertainty in their position. As such their position will form a distribution of states that will lower the absolute intensity of the signal, thus reducing the absolute distance measured, and increasing the need to minimise errors in measurement. Use of stiffer linkers will ameliorate much of this, although the behaviour will still be a feature of longer DNA fragments. However, such information on average distances can be used to inform molecular dynamics studies of protein/DNA complexes: such an approach should be quite fruitful for future research.

## Conclusions

The theoretical work presented shows that it is possible to use anomalous SAXS and nanocrystal labels attached to biomacromolecules to measure distances. In addition, more specific distance information can be extracted using nanocrystals of different metal types. After accounting for likely errors in the system, and taking into account the range of energies available at today's synchrotron sources, it should be possible to determine the end-to-end distance of a molecule like DNA to near-Angstrom resolution. Our simulations used a gold nanocrystal containing exactly 78 atoms, however increasing this size will obviously increase the signal-to-noise ratio, but decrease the resolution with which we can determine the distances. However, with correctly designed experiments, homogeneous samples and good set-up on an appropriate beamline, ASAXS will be able to derive valuable information on molecular distances in biomacromolecular complexes.

## Supporting Information

Figure S1
***Partial structure factor*** **.** Gold nanocrystal partial structure factor 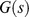
for a 50 base-pair DNA molecule before truncation and baseline shifting. The oscillatory nature of the function is clear at small *s* values, but an increase in the contribution of random errors can be seen as *s* increases. This data was truncated at 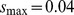 Å^−1^ where the oscillations are almost indistinguishable from the noise.(TIFF)

Programs S1 **MATLAB codes.** A zipped file containing the MATLAB code files used in this work. The code runs through a graphical user interface, which can be started with the command “saxs_label”.
(ZIP)
