# Supplementary figures and images for: Anomalous Small Angle X-Ray Scattering Simulations: Proof of Concept for Distance Measurements for Nanoparticle-Labelled Biomacromolecules in Solution
Source: PLoS One. 2014 Apr 23;9(4):e95664. doi: 10.1371/journal.pone.0095664 (PMC3997412; doi:10.1371/journal.pone.0095664)

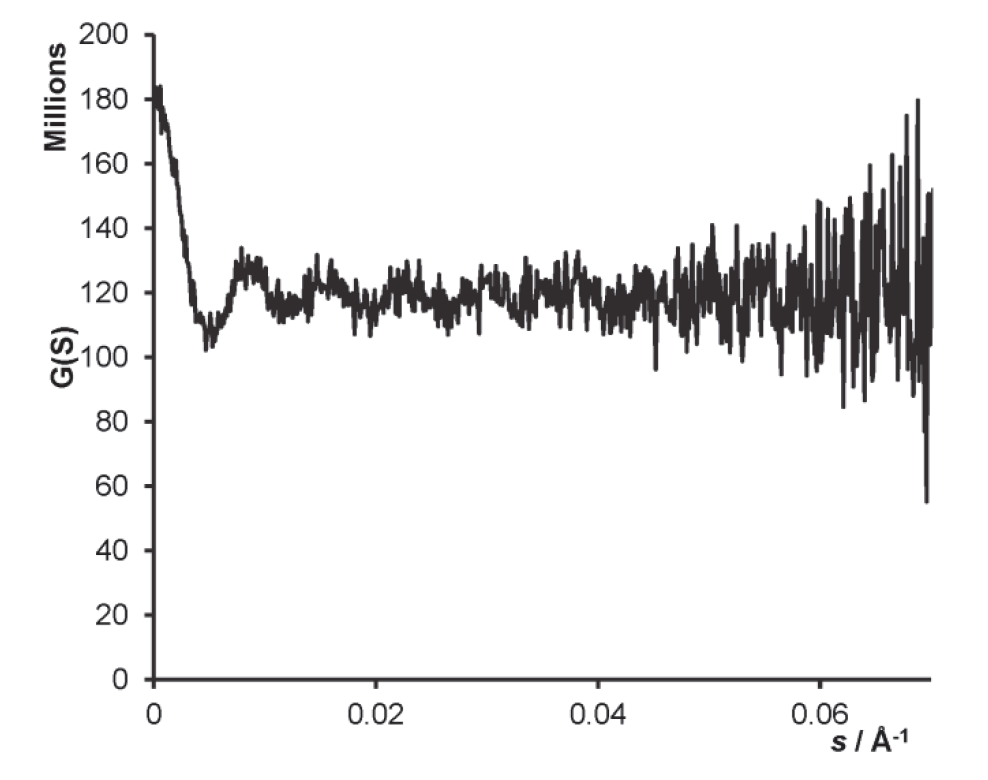

Supplement: Figure S1 — Partial structure factor . Gold nanocrystal partial structure factor for a 50 base-pair DNA molecule before truncation and baseline shifting. The oscillatory nature of the function is clear at small s values, but an increase in the contribution of random errors can be seen as s increases. This data was truncated at Å−1 where the oscillations are almost indistinguishable from the noise. (TIFF) [file pone.0095664.s001.tiff]
